# The Prediction of Peritoneal Carcinomatosis in Patients with Colorectal Cancer Using Machine Learning

**DOI:** 10.3390/healthcare10081425

**Published:** 2022-07-29

**Authors:** Valentin Bejan, Elena-Niculina Dragoi, Silvia Curteanu, Viorel Scripcariu, Bogdan Filip

**Affiliations:** 1 Department of Surgery, Faculty of Medicine, University of Medicine and Farmacy “Gr. T. Popa” Iasi, 700115 Iasi, Romania; viorel.scripcariu@umfiasi.ro (V.S.); bogdan.filip@umfiasi.ro (B.F.); 2 Faculty of Chemical Engineering and Environmental Protection, “Gheorghe Asachi” Technical University of Iasi, 700050 Iasi, Romania; elenan.dragoi@gmail.com (E.-N.D.); silvia_curteanu@yahoo.com (S.C.)

**Keywords:** peritoneal carcinomatosis, colon cancer, rectal cancer, neural networks, differential evolution algorithm

## Abstract

The incidence of colon, rectal, and colorectal cancer is very high, and diagnosis is often made in the advanced stages of the disease. In cases where peritoneal carcinomatosis is limited, patients can benefit from newer treatment options if the disease is promptly identified, and they are referred to specialized centers. Therefore, an essential diagnostic benefit would be identifying those factors that could lead to early diagnosis. A retrospective study was performed using patient data gathered from 2010 to 2020. The collected data were represented by routine blood tests subjected to stringent inclusion and exclusion criteria. In order to determine the presence or absence of peritoneal carcinomatosis in colorectal cancer patients, three types of machine learning approaches were applied: a neuro-evolutive methodology based on artificial neural network (ANN), support vector machines (SVM), and random forests (RF), all combined with differential evolution (DE). The optimizer (DE in our case) determined the internal and structural parameters that defined the ANN, SVM, and RF in their optimal form. The RF strategy obtained the best accuracy in the testing phase (0.75). Using this RF model, a sensitivity analysis was applied to determine the influence of each parameter on the presence or absence of peritoneal carcinomatosis.

## 1. Introduction

Colon, rectal, and colorectal cancers (CRC) are some of the most commonly diagnosed cancers worldwide: fourth, eighth, and third, respectively. Colorectal cancer is also the second cause of cancer mortality [1]. About one-third of all patients are diagnosed in the advanced stages [2] because of myriad heterogenous etiological factors: the lack of specific, easily recognizable symptoms, and delayed diagnosis and treatment [3,4]. Regarding etiology, genetic factors account for only 10% of cases, with the rest resulting from the interaction between environmental factors and nonhereditary events. Consequently, identifying a high-risk population subgroup is difficult [5].

Peritoneal carcinomatosis (PC) involves the peritoneum and can occur as a late-stage manifestation of gastrointestinal tumors. Historically, these patients were given palliative care [6], but now there are aggressive therapeutic options (e.g., cytoreductive surgery with or without intraperitoneal chemotherapy). Unfortunately, there are limited cancer research centers where these treatment methods can be performed, usually on selected patients with minimal peritoneal burden [7]. To date, no diagnostic tool can identify early peritoneal carcinomatosis in patients with colorectal cancer. When the peritoneal burden is high, imaging tools can only identify peritoneal dissemination in advanced stages.

Early suspicion of carcinomatosis through machine-learning models using routine blood tests would enable better patient management and lower mortality and morbidity [8].

The study aimed to use various machine learning strategies, artificial neural networks (ANNs), support vector machines (SVM), and random forests (RF) methods to increase the prediction of PC using basic blood parameters so that patients could be referred to specialized centers more quickly. In order to automatically fine-tune the parameters of each machine learning (ML) strategy, the differential evolution (DE) algorithm was applied as an optimizer. Multiple ML strategies were applied to efficiently determine the best-suited approach to classify the considered cases based on the available parameters.

ANNs are inspired by the biological brain and have an excellent ability to map nonlinear inputs-outputs relations. Despite their effectiveness and easiness of use, the optimal configuration of an ANN is not easy to set up. On the other hand, SVMs have a good generalization capability and a reduced number of parameters to identify compared to ANNs. RF are ensembles of tree predictors where the generalization error is dependent on the performance of each individual tree and the correlation between them.

The applied ML methods (ANN, SVM, and RF) are well-known algorithms. However, few examples are known in which they have been associated with an evolutionary algorithm (DE) as an optimizer for parametric and structural determination. In addition, the novelty of this research related to the application of these tools for the prediction of peritoneal carcinomatosis is worth mentioning.

The paper of Ramesh et al. [9] presents an overview of different artificial intelligence techniques applied for diagnosis, treatment, and outcome prediction in many clinical scenarios. The most often used tools in clinical settings are: ANNs, fuzzy expert systems, evolutionary algorithms, and hybrid intelligent systems. This general medical application provides a global vision of the possibilities of simulations and the results that can be obtained using artificial intelligence tools.

An interesting mini review contained some general statements about applying ANN in medicine. The paper classified how neural networks have been applied based on the type of data used to train the networks. Applications of neural networks to medicine can be categorized into two types: automated diagnosis and physician aids. More precisely, the application of neural networks trained with medical images and data from medical records is presented here [10].

In the approach of Sabuj and Biswas [11], the colon cancer stages were defined using the TNM classification, in which T defines tumor characteristics; N refers to lymph node involvement; and M stands for metastasis (Table 1). Each stage included a particular tumor grade, specific histology, tumor location, number of positive lymph nodes, and metastases as input data. Feedforward neural networks provide accurate results in classification tasks such as tumor staging, diagnosis, or survival prediction.

A microarray study [12] was conducted to evaluate the ability of an ANN and hierarchical cluster analysis to discriminate between two types of cancer––sporadic colon adenomas (SACs) and inflammatory bowel disease-related dysplasia (IBDNs)––based on hybridizing 8064 cDNA clones to mRNAs derived from 39 neoplastic colon specimens. Whereas ANN correctly diagnosed 12 of the 12-blinded samples, the hierarchical analysis failed because of noise in the data.

In another approach [13], a combinatorial selection method in conjunction with an ensemble neural network was applied to analyze cancer data, including colon cancer. The main idea was to combine the best features of the two instruments to choose top-ranked genes that would give more information and combine the output of several neural networks from an aggregate to give network stability and a robust answer. Good results were obtained with a predictive accuracy of over 90%, even with increased computational complexity and additional time needed to perform the analysis.

The applications of three-layer feedforward ANNs with backpropagation error contributed to improved colon cancer classification and survival prediction accuracy compared to other statistical or clinic-pathological methods [14]. In addition to the examples described, there are several general elements related to using neural networks in biomedical applications concerning their advantages, disadvantages, and particularization in colon cancer. The main advantages are: (a) a requirement for less formal statistical training; (b) better-discriminating power than other regression models; (c) trainable with noise-affected data; and (d) the ability to detect complex nonlinear relationships among variables.

Their disadvantages include: (a) the inability to understand interactions among variables because it is a “black box” model; (b) limited ability to identify possible causal relationships; (c) empirical model development; (d) models prone to overfitting; (e) significant time and resources needed for model design; and (f) a considerable amount of data needed for neural model development and training.

It is necessary to conclude that feedforward neural networks contribute to colon cancer’s improved diagnosis and prognosis, despite the difficulties encountered.

Different deep-learning ANNs were used to obtain predictions for survival and conditional survival for colon cancer over 1, 2, and 5 years [15]. The authors reported an approximate 0.87 area under the receiver operating characteristic curve measurements. Two baseline classifiers, random forests and logistic regression, were used for comparison and other previous works emphasized the superior performance of neural modeling.

A partial logistic artificial neural network was developed and trained to predict cancer-related survival in patients with confirmed colorectal cancer. This model was validated against Kaplan–Meier observed survival plots of a random sample of 300 patients not used in the training phase. Close agreement between the two sets of data proved the reliability of the neural model [16].

Predicting outcomes for colorectal cancer patients following surgery is essential as almost 50% of patients undergoing a potentially curative resection will experience a relapse. Two analysis methods, logistic regression and neural networks, were applied to model disease recurrence, using data from 403 patients [17]. Their results were compared with receiver–operator characteristic plots that estimate the model’s fit. The best logistic regression model gave a result of 66%, and the neural network approach gave a result of 78% [17].

Another approach was developing an ANN model to predict survival after liver resection for colorectal cancer metastases. The prognostic factors included in the model were age, preoperative chemotherapy, size of the most extensive metastasis, hemorrhagic complications, preoperative CEA level, and the number of metastases. The C-index was 0.72 for the ANN model and 0.66 for the Cox regression [18].

In an interesting study [19], the researchers aimed to develop and validate an ANN model to predict post-hepatectomy early recurrence (PHER) in hepatocellular carcinoma (HCC) patients without macroscopic vascular invasion. This was motivated by the fact that accurate prediction of PHER of HCC is vital for determining postoperative adjuvant treatment and monitoring. Nine hundred and three patients who underwent curative liver resection for HCC participated in this study. They were randomly divided into derivation (n = 679) and validation (n = 224) cohorts. The ANN model was developed in the derivation and verified in the validation cohort. The main conclusion was that the ANN model had a significant advantage in predicting PHER for HCC patients without macroscopic vascular invasion compared to other models and staging systems.

It seems that neural networks are gaining more and more ground, becoming valuable tools, along with clinical observations and paraclinical investigations, to predict and diagnose colorectal cancer in various stages and with different distinctive traits. Therefore, the study’s main objective was to evaluate if ANNs, SVM, and RF can be reliably used to predict early PC in patients diagnosed or with a high probability of having colorectal cancer. In addition, age, sex, and six routine blood parameters (platelets, white blood count, hematocrit, hemoglobin, neutrophil, and lymphocyte count) were used as input data, thus increasing the availability of the method and enabling a multilateral approach.

## 2. Materials and Methods

### 2.1. Data Gathering and Processing

A retrospective study was performed using data from patients admitted to the first and second surgery clinic at Sf. Spiridon Hospital in Iași, Romania, between 2010 and 2020. The data were anonymized and confidential and consisted of routine blood tests. The study was conducted following the Declaration of Helsinki, and the protocol was approved by both the Ethics Committees of the Sf. Spiridon Hospital and of the University of Medicine and Pharmacy” Grigore T. Popa” Iași.

**Figure 1:**
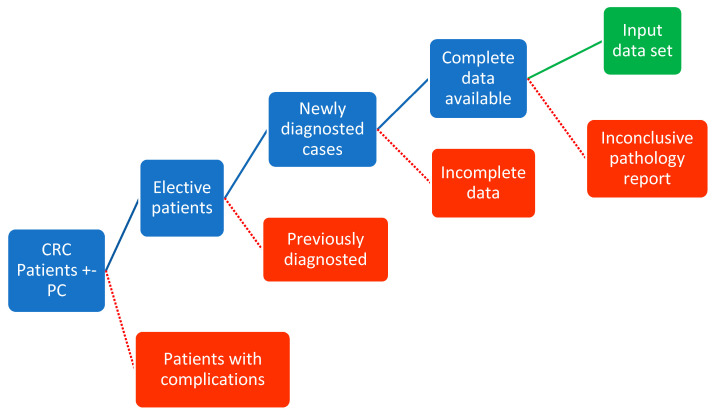
Flowchart of patient inclusion and exclusion algorithm.

Rigorous inclusion or exclusion criteria were applied to the initial data pool (Figure 1). All patients priorly diagnosed with PC or referrals with a probability of a high PC burden were excluded, as were patients with CRC complications (intestinal obstruction, peritumoral abscess) or any other underlying condition such as concomitant infections that would influence the blood panel. Incomplete data sets were also excluded. The CRC diagnosis was established through a pathology report. Despite suggestive clinical and paraclinical findings, patients with inconclusive pathology results were excluded, as were patients with appendicular mucinous carcinoma due to its rarity and particular pathophysiology. Pathology reports were also excluded from the data sets. While these could have provided additional information that might have improved predictions, the use of reports would have undermined the initial goal of the study; the same reasoning was applied to imaging and tumor markers. Patients included in the study were diagnosed with CRC and PC during their current admittance. In all cases, a PC diagnosis was solely an intraoperative finding, which accorded with the study’s aims. In all cases, the peritoneal burden was low.

The parameters in the database considered significant for this approach were: sex, age, hemoglobin levels (Hb), hematocrit (Ht), platelet count (PLT), white blood cell count (WBC), neutrophils (Neutr), and lymphocyte counts (Lymph). In the absence of any described blood marker or panel that can be correlated with this natural evolution stage of intraabdominal cancers, we have chosen these parameters to consider the different systems affected by cancer progression. Taken separately, many diseases can determine the normal pathological range, thus making them highly nonspecific. Using a panel of 6 blood tests, we have attempted to improve specificity by using distribution patterns described by our patient data sets and supported directly or indirectly by medical literature data. Considering cancer is a systemic disease, although originating and initially progressing locally, we try to improve specificity by altering these parameters (hematopoiesis and inflammation, which can be considered altered in one way or another in all cancer patients). Regarding sex and age, incidence patterns are better described than other parameters. This choice falls within the scope of the study: to diagnose PC using machine learning and routine blood tests, thus increasing accuracy and availability.

After the data was gathered and analyzed, a verification procedure was applied. This entailed removing data for which not all analyzed parameters were measured and conducting a standard statistical analysis to identify outliers and determine whether transcription errors occurred during database compilation. The final dataset consisted of 95 patients. For the current study, we used the following input parameters or predictors (n = 8): sex, age, Hb, Ht, PLT, WBC, Neutr, Lymph, and the number (N), and one output variable: the presence or absence of peritoneal carcinomatosis. The minimum, maximum, mean, and standard deviation of the predictors are presented in Table 1 and Table 2. 

To determine if there are differences between the parameters of the two groups, the two-sample t-test was applied using Minitab. The results obtained had a *p*-value < 0.05 for age (*p* = 0.003), Hb (*p* = 0.003), and Ht (*p* = 0.005), pointing out that for these three parameters, there is sufficient evidence to indicate significant differences between the two groups. For the other parameters, the *p*-value was >0.05, as follows: *p* = 0.182 for sex, *p* = 0.961 for HLT, *p* = 0.259 from WBC, *p* = 0.163 for Neutr, and *p* = 0.617 for Lymph.

As PC represents a local spread of the CRC, it did not alter homeostasis specifically. With these considerations, the initial patient groups comprised a small, relatively homogenous data pool, which could justify low precision and recall.

Table 1 depicts the group diagnosed with colorectal cancer and peritoneal carcinomatosis. Table 2 represents the patients diagnosed only with colorectal cancer. The first difference between the groups is related to age. Patients diagnosed with PC were eight years younger than those in the control groups. This was attributed to the aggressiveness of the tumor. The patients in the first group also had an upper limit or higher WBC and neutrophile count, reinforcing the link between cancer and immunity. The platelet count in both cases was normal, but it reached the upper limit in the second group, which had greater data dispersion.

To put all parameters into numerical form, sex was coded 1 for male and 2 for female, and to improve the efficiency of the ML approaches, three standard processing techniques were applied. The first was “randomization”, where the entire dataset was randomly re-arranged. The second was “label assignment”: the first 75% of the data were included in the training dataset, which was used to determine the characteristics of the ML approach that best classified colorectal cancer occurrence; the remaining 25% was assigned for testing. The rationale behind randomization and label assignment is to eliminate the probability that the considered approaches learn from a reduced number of similar examples, which can significantly influence its generalization capability. The third technique was “normalization”, where the range of each input was constrained within the 0.001–0.999 interval using a min-max approach [20].

### 2.2. Classification 

**Figure 2:**
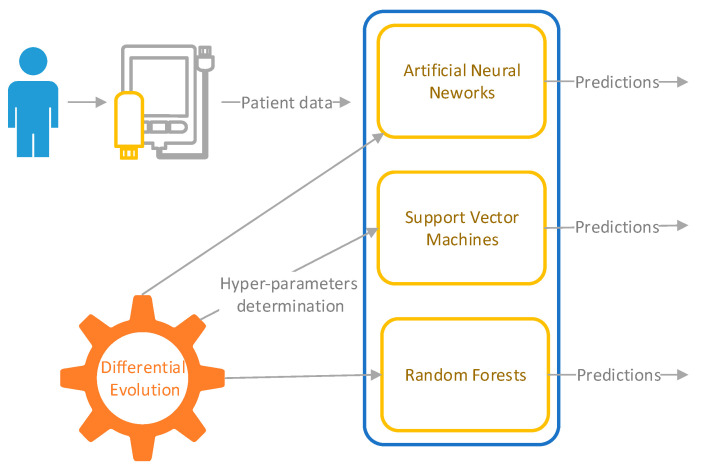
Workflow for data classification.

To determine the presence or absence of peritoneal carcinomatosis of colorectal origin based on sex, age, Hb, Ht, PLT, WBC, Neutr, and Lymph, a methodology including three ML strategies (ANNs, SVM, and RF) combined with DE was applied. The simplified workflow of the ML-based classification performed in this work is presented in Figure 2.

ANNs are computational structures that mimic the functioning of the brain. Although there are significant differences in complexity between the biological neural networks and its counterpart, the learning, adaptive mechanisms, and computational effectiveness of ANNs proved efficient for various problems [21,22]. Their main advantages are the ability to deal with uncertainty, approximate any continuous nonlinear relation, and self-learn. For classification problems, they also performed better than the classical approaches [23].

An ANN is formed from interconnected processing elements called “artificial neurons” and the number and organization among them represent its topology (or “architecture”). On the other hand, the type of neurons and their interconnections indicate the type of ANN. The most widely known and used type of ANN in classification and regression problems is the feedforward multilayer perceptron (MLP) [24], which has the following characteristics: the neuron type is a perception; neurons are organized in layers; and each neuron from one layer is fully connected to the neurons from the next layer. Furthermore, information flows from inputs to outputs without loops or recurrent connections. In addition to these features, an ANN also has a series of internal parameters that are either fixed, manually set, or modified during the training procedure that efficiently classifies a given problem. For this study, a sequential model with dense layers, Relu activations in the hidden layers and sigmoid activation for the last layer (parameters that need to be identified for the excellent functioning of the model), and Adam optimizer (the strategy for optimizing the training process) was considered.

Although ANNs are simple to use, determining the optimal architecture and parameters is a difficult task for the two following reasons: (i) the optimal topology and internal parameters are dependent on the characteristics of the problem being solved; and (ii) the literature does not provide rules that can be applied generally for all types of problems. In this context, the optimal ANN determination in the current work is performed by combining it with DE. This combination is a particular example of neuro-evolution, an area where various optimizers evolve ANNs to eliminate the difficulties specific to their manual determination. Consequently, the optimizer (DE in our case) determines structural parameters that define an ANN in its optimal form.

Similar to ANNs, the SVM and RF approaches have a series of parameters that must be optimally identified to have good results. For example, SVMs perform better when there is a clear separation between the classes and a high-dimensional space. On the other hand, they do not perform well when the available data is noisy. For the SVM, if the data training is not linearly separable, their performance is not good. Thus, the input vectors must be transformed into vectors of a high dimensional feature space using a kernel function. The most used kernel functions are linear, polynomial, Gaussian, RBF, and sigmoid. Another parameter considered for optimization in the case of SVM is represented by C, the cost or penalty parameter. 

Like ANNs and SVMs, RF can be used for regression and classification problems. However, RFs are not influenced by outliers and usually provide high accuracy. Nevertheless, RFs are challenging to interpret and can be computationally expensive when used for large datasets. In the case of RF optimization, the number of estimators is the only parameter considered

DE, a metaheuristic inspired by the Darwinian principle of evolution, was chosen for the current case study for its advantages (such as simple structure and reduced number of parameters) and effectiveness. Although initially designed for complex optimization problems over continuous spaces, DE is a universal algorithm that can be applied (in a simple or in modified form) to almost any type of problem [25].

In medicine, among others, it has been applied to early breast cancer detection [26] and optimizing targeted drug delivery to treat tumors [27]. In combination with ANNs, DE was also used to classify clinical datasets (diabetes, breast cancer, and heart disease) [28] and to segment brain tumors [29].

Like all evolutionary algorithms, DE evolves an initial population of solutions using operations such as mutation, crossover, and selection. Then, the process is repeated until a stop criterion is reached. In the current work, the role of DE is to optimize the ANN from a structural point of view (number of layers and neurons in each hidden layer) and to identify the most critical parameters for SVM and RF.

The optimization procedure starts with initialization. This step entails randomly generating potential solutions. An opposition based learning (OBL) [30] principle was included to improve this step. To achieve a better approximation, OBL compared an individual with its opposite. There were various OBL variants [31], and, in the current work, the classical approach proposed by Tizhoosh was used [30].

After that, the mutation step is applied, which consists of generating a population of mutants through the application of a differentiation operator (Equation (1)) on the current population:

(1) $$\omega_{i}=\alpha +F\cdot \beta$$

where *ω_i_* is the *i*th element from the mutated population; *α* is the base vector; *β* is the differential term; and *F* is the scaling factor, one of the control parameters of DE. The differential term is the difference between two or more distinct, randomly chosen vectors: $\beta =x_{k}-x_{p}$.

In the next step, crossover, new individuals are created based on the current and mutated population. The DE algorithm generally uses two crossover variants: binomial and exponential. However, the binomial variant (Equation (2)) is applied here due to its advantages.

(2) $$u_{i,j}=\{\begin{array}{c} \omega_{i,j}\;\;if\left(rand\left(0,1\right)<Cr\right)\\ x_{i,j}\;\;\;\;\;\;otherwise \end{array}$$

where *u_i_* is the trial vector resulting from crossover application and *x_i_* is the *i*th individual from the current population. Finally, *Cr* is another control parameter of DE.

In the final step, selection, the trial population is compared with the initial population, and the best-suited individuals are selected to participate in the next generation. The measure used to determine the performance of each individual is called “fitness”. In the current approach, because the individuals represent encoded ML parameters, fitness was based on the mean square error (MSE) for ANNs, and accuracy for SVM and RF.

Usually, the DE control parameters *F* and *Cr* are set manually. However, as their values are problem-dependent and strongly influence the algorithm’s performance, in this work, a self-adaptive procedure for the control parameters was included in the algorithm itself. 

The implementation of the entire classification methodology previously described was performed in Python. The complex mathematical operations were implemented using the NumPy library, and the ML approaches combined with the DE strategy used TensorFlow and scikit-learn libraries. The simulations and tests to determine the efficiency of the proposed approach were performed on a computer with an Intel I9 processor, 16 GB RAM, NVIDIA Quadro P2000 video card, and a 512 GB PCIe NVMe hard disk.

### 2.3. Sensitivity Analysis

When modeling a specific problem, a key question is related to the importance of inputs. One method that can be used for such a task is sensitivity analysis (SA). For example, it can rank inputs based on their influence on the output to improve quality by assessing output changes based on input variations or limiting program use [32]. Since the implementation of all the approaches considered in this work used Python, Tensorflow, and Sklearn, the sensitivity analysis and feature evaluation was performed using the specific functions available in the used frameworks. In the case of RF, for example, the feature_importances_ attribute of the RandomForestClassifier model indicates the feature importance.

## 3. Results 

### 3.1. Dataset Analysis

The input data are sex, age, and laboratory findings such as Hb, Ht, PLT, WBC, Neutr, and Lymph. These parameters reflect different pathophysiological aspects of cancer and are usually found outside regular intervals for these patients. Age and sex heavily influence all aspects of cancer: incidence, type, aggressiveness, prognosis, morbidity, and mortality. A paraclinical feature in most cancer patients is anemia, represented in our database by Hb and Ht. The link between cancer and inflammation is now considered a certainty, although the exact interactions and results are unquantifiable. WBC, Neutr, Lymph. and PLT represent different aspects of the immune system and are usually modified in cancer patients. Another important reason for selecting these laboratory findings is that they are part of the standard blood work for every patient admitted, regardless of the clinic’s specialty. This makes them an ideal prediction factor because they are widely available and low cost.

### 3.2. Classification Results

Before applying the ML strategies, a series of classical statistical approaches were tested to assess if the considered case study could be solved using traditional methods. Thus, the Minitab software was used to determine regression models. In order to transform the continuous output into a categorical one (and to better compare with the ML-based classification strategies) the regression predictions were rounded to the nearest integer. From a total of 95 exemplars, 38 and 50, respectively, were incorrectly classified by the results of the full factorial regression model and the forward selection model. This high error rate indicates that classical regression models are unsuitable for the considered problem.

Thus, to determine a good ML classification model, the procedure combining ANN and DE was applied first, using the following setting: the number of generations (iterations) was 100, and the number of individuals in the population was 30. Regarding the maximum allowed topology, because it is closely related to the number of parameters encoded into the structure DE works with, a limit was imposed on the number of hidden layers and neurons in each hidden layer. These limits were based on preliminary tests and the authors’ experience. It consisted of a maximum number of allowed hidden layers (5), with 50, 30, 30, 30, and 30 neurons maximum in each hidden layer.

The best solution found had a dense hidden layer with 10 neurons and a dense output layer with a softmax activation function with 2 neurons. Since the softmax activation function provides a list of probabilities for the ANN, the 1 output classification was transformed into a 2-output problem. The total number of trainable parameters for the best identified ANN model was 112. The trained model has a loss value of 0.1793 in the training phase. The number of misclassified exemplars is 12 in the training and 15 in the testing phases. As the MSE is a good indicator for regression and, as in our case, a classification of cancer presence was performed, the following indicators were computed for the best ANN: True Negative (TN), True Positive (TP), False Negative (FN), and False Positive (FP) organized into a confusion matrix (Table 3) for both training and testing data.

**Figure 3:**
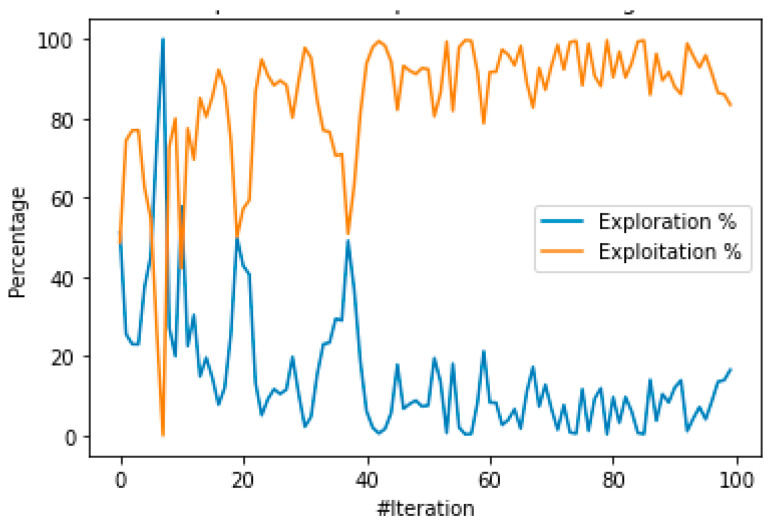
Exploration exploitation balance of the DE algorithm.

In the case of the SVM optimization, the optimizer had a good balance between the exploration and the exploitation phases, as seen in Figure 3. This allowed an excellent performance in identifying reasonable solutions; the confusion matrix obtained is presented in Table 4.

The only parameter that varied for the RF optimization is the number of estimators. The solution provided by the DE algorithm was 150; the confusion matrix is presented in Table 5.

As it can be observed, the best strategy with the lowest misclassification rate is the RF approach, for which, in the training phase, there were no classes misclassified. The overall statistics obtained with all the strategies for the testing data are presented in Table 6.

### 3.3. Sensitivity Analysis Results

Using the best model determined previously using the RF approach, the sensitivity analysis provides the importance of each feature considered as input for the developed models (Table 7).

## 4. Discussion

Artificial intelligence techniques were applied to determine the influence of sex, age, hemoglobin (Hb), hematocrit (Ht), platelet count (PLT), white blood count (WBC), neutrophile (Neutr), and lymphocyte (Lymph) count on the occurrence of PC in patients with colorectal cancer. The objective was to determine a classifier that considered all the possible interactions and to identify if PC were present or not. To these means, the tool chosen in this work was presented using different ML approaches and differential evolution (DE) as a model optimizer.

Regarding the sensitivity analysis, the higher the values from Table 7, the higher the influence of that input on the model’s output. Thus, the most minor important two parameters were PLT and sex. The influence of the other parameters was, to some extent, confirmed by existing scientific data. As such, although CRC incidence is more often found in male patients, the presence of PC is more dependent on factors other than sex. The input parameters with higher sensitivity coefficients were age, Ht, and Hb, mirroring a more significant immunological burden.

### 4.1. The Link between Sex, Age, and Colorectal Cancer

Worldwide, males have the highest incidence of colorectal cancer and are about 1.5 times more likely to develop it. However, by comparing different epidemiological studies regarding geographical areas and countries, a very heterogenous incidence pattern unfolded with significant variability between developing and developed countries and among different geographical areas. For example, some countries (e.g., Saudi Arabia, United Arab Emirates, and Oman) reported colorectal cancer as the first cause of cancer-related mortality in the male population. In other countries (e.g., Algeria, Belarus, Japan, Spain, and Portugal), it represented the leading cause of female-related cancer [33]. Although these dissimilarities exist regarding mortality, there is no instance where the incidence between the sexes is reversed.

Most cases are diagnosed after the age of 50. Colorectal cancer (CRC) in young adults is characterized by poorly differentiated, more aggressive histological types, with almost the same incidence between the sexes. This can be explained by the more significant role of hereditary factors. However, certain environmental factors such as obesity, nutritional factor, and sedentary lifestyle have been incriminated for a rising incidence of CRC in patients <50 years old [34]. Regarding localization, ascending colon cancer is more frequent in female patients. Usually, these types of neoplasia exhibit ambiguous or no symptoms in the early stages; therefore, diagnosis occurs in the advanced stages of the disease. This would explain female colon cancer patients’ higher mortality and lower 5-year survival rates [1].

A study of the relationship between sex, hormonal and reproductive factors, and microsatellite instability (MSI) confirmed the protective influence of estrogen in the development of MSI, thus explaining the greater incidence of aggressive MSI-high cancer in postmenopausal patients. To further confirm this theory, patients who followed hormone-replacement treatment only rarely presented genetically unstable types of CRC, the majority being in the more favorable differentiation types or stages [35,36]. However, the beneficial role of estrogen is still contested despite these findings. At first glance, it plays a protective role in CRC development, but once the oncogenic process has begun, it facilitates proliferation and tumor growth. This dual role could provide a future cause for the high mortality rate in female patients over 65 [37].

Due to the multifactorial ethology of CRC, studies were undertaken to identify the causes of the different incidences between the sexes. Therefore, the first and most logical approach was to identify genetic or epigenetic factors. Thus, specific mutations were identified more commonly in female CRC patients (such as *PIK3CA*) or exclusively in these patient subgroups (VEGF polymorphism, variant 936, or the methylation in the 5′ position of the tumor suppressor gene *p*16*^1NK4a^*) [38,39,40].

Caution is warranted since there exist only a few preclinical studies of the genetic and molecular mechanism involved in carcinogenesis using test animals of both sexes. In addition, epidemiological studies are necessary to identify further the biological variables linked to CRC incidence and mortality.

### 4.2. Anemia-Cancer

Newer definitions of anemia have a more qualitative than quantitative approach, focusing on the inability to perform adequate oxygen transport to the capillaries. There are numerous causes of anemia, ranging from an absolute decrease in the number of erythrocytes to a quantitative or qualitative deficit in hemoglobin.

Although cancer is a hypernym for multiple conditions associating diverse and complex manifestations from a common etiopathogenic event (carcinogenesis with DNA mutations in critical regions responsible for cellular division), anemia is one of the common features. 

It is estimated that somewhere between 40% and 67% of all cancer patients will have anemia, either at the moment of diagnosis or during the evolution of the disease [41]. According to ECAS, the most extensive study of anemia in cancer, 39% of all CRC patients had it at the point of diagnosis and 61% presented with different degrees of anemia during the study. However, anemia treatment was initiated for values of Hb under 9.5 g/dL; therefore, only 31% of patients received this treatment [42].

Multiple factors contribute to the onset of anemia: tumoral hemorrhage, hemolysis, hypersplenism, renal failure associated with decreased erythropoietin production, bone marrow failure due to metastasis, myelodysplastic syndrome, or chemoradiotherapy-associated myelotoxicity [43]. The mechanisms described above do not fully explain anemia in cancer patients. Clinical observation and in vitro studies have begun to uncover the link between the cancer trigger response in the host immune system and anemia. A cell-mediated immune response exists through unknown mechanisms, resulting in increased cytokine production via activated T lymph cells and macrophages. The cytokine-mediated effects include inadequate differentiation and proliferation of erythroid progenitor cells, impaired normal iron metabolism by decreasing circulating levels via trapping in the endoplasmic reticulum, decreased erythropoietin production, and possibly shortening of the erythrocyte lifespan [44].

### 4.3. Immune System—Cancer Interaction

Rudolph Virchow, who first observed white blood cells inside cancer specimens through the microscope 150 years ago, first theorized about the existence of an interaction between the host immune system and cancer [45]. However, only in the last decades, primarily through in vitro studies, specific intimate, sometimes surprising, mechanisms of this interaction have been deciphered. 

Observational epidemiological studies revealed a link between inflammatory bowel disease (an autoimmune condition) and the increased risk of colorectal cancer, thus incriminating an immune system-related factor [46]. The tumoral microenvironment also represents an essential step in carcinogenesis and tumor development through yet not fully understood mechanisms. 

Alongside its possible role in carcinogenesis, the immune system is involved in tumor growth, metastasis, and conventional chemotherapy resistance. These actions are enabled through the tumor’s subversive activation of immune cells (T lymphocytes and macrophages) and, consequently, the production and release of cytokines [47].

To evaluate the function of the immune system, the most commonly used tests are the total numbers of leukocytes/mm^3^, lymphocytes, and neutrophils, representing 60–70% of circulating white blood cells. Based on animal studies, these cells mature in the bone marrow, and only 1–2% will become circulating cells [48]. In cancer cells, there are alterations to this type of leukocyte. Qualitatively, the life span increases from 7 to 17 h, and variable degrees of neutrocytosis can be observed, thus mirroring the host immune response. However, their exact role in tumor development is to be uncovered. Yet, neutrophil degranulation with the release of cytokines, free oxygen species, and angiogenetic factors is theorized, as is local immune system modulation.

Another parameter proposed to describe better the magnitude of this specific immune response is the neutrophil-to-lymphocyte ratio (NLR). An increase in the number of neutrophils (considered promoters of cancer development) and an associated decrease in the number of lymphocytes (with their postulated role in tumor suppression) is easier to investigate. An increased NLR value is associated with a worse prognosis and weaker response to treatment [49,50].

Another inflammatory marker is platelets. Unlike neutrophils (involved in the first phases of inflammation) and lymphocytes (which provide specific, cell-mediated immunity), these are nonspecific markers representing the turning point in hemostasis–inflammation–tissue repair. Once activated, they exhibit receptors on their surface for adhesion molecules on the endothelium, granulocytes, lymphocytes, and monocytes. Reactions on receptor binding release cytokines and other inflammatory mediators, amplifying or reducing the immune response [51]. Similar to NLR, a platelet-to-lymphocyte score was proposed, with a similar result interpretation. 

However, the usefulness of these parameters is controversial. Up-to-date studies present a high degree of heterogeneity, with a lack of consensus regarding cut-off points and important variables concerning the total number of white blood cells, geographical area, and ethnicity [52].

The study had some limitations. Most important was the low number of cases due to the rigorous inclusion criteria. By acknowledging that cancer is a consumptive disease, eliminating the data from patients with advanced stages or complications resulted in a data set that accurately matched the aim of the study: Can an ML model predict early-stage PC? The proposed model predicted PC in more than 80% of CRC patients. Although not ideal, this approach might be a valuable diagnostic aid after refining the input data and training with larger datasets.

Another limitation was perhaps the blood panel representing the input data. Although nonspecific, alterations of these parameters mirrored the etiopathogenesis and evolution of cancer. Nevertheless, the main reason for choosing the specific parameters was that all were part of the standard blood panel. Considering the rate of software development, adding an ML model to the central patient database would greatly benefit both the patient and the health care system through faster specialist referral and reduced overall costs. Even primary care would benefit, considering that certain types of CRC can be suspected through a thorough clinical examination and digital rectal examination. With a prediction rate of 80% as in the present study, patients would be more easily referred to secondary and tertiary care centers, thus reducing mortality, morbidity, and overall cost in these cases.

## 5. Conclusions

This article reviewed several approaches based on artificial intelligence for colorectal cancer predictions, particularly ANNs, SVM, and RF. In addition, several theoretical considerations were made regarding what is known about possible factors that would influence disease evolution: sex, age, dietary factors, anemia, and immune system. 

The study’s objective was to develop ML models associated with the DE algorithm to increase the prediction of PC using basic blood parameters to enable easier patient reference to specialist centers. These models were represented by artificial neural networks, support vector machines, and random forest tools, whose parameters were optimized with an evolutive technique.

To date, there are no studies in the literature regarding any type of blood test or test panels able to diagnose the presence of PC. In cases with a low peritoneal burden, patients are more likely to receive and benefit from aggressive multimodal treatment, the diagnosis of which is usually set intraoperatively. Imaging diagnostic tools can usually diagnose PC with a medium-to-high peritoneal burden, depending on the technology used, yet are rarely able to detect incipient PC. The RF-DE was also applied for the first time to predict colorectal cancer.

Finally, the results are susceptible to improvement, but their merit is that they open a promising study niche with important practical implications for people’s lives.

## Figures and Tables

**Table 1:** Statistics of the parameters considered for carcinomatosis and colorectal cancer study group.

	N	Minimum	Maximum	Mean	Std. Deviation
Sex	46	1	2	1.41	0.50
Age (years old)	46	36	84	62.22	11.50
Hb (g/DL)	46	6.5	16.5	12.20	2.66
Ht (%)	46	19.9	49	37.18	7.00
PLT (/mm^3^)	46	152,000	702,000	339,347.83	112,512.96
WBC (/mm^3^)	46	2830	85,700	11,187.37	11,761.72
Neutr (/mm^3^)	46	10.07	64,700	9082.39	11,336.75
Lymph (/mm^3^)	46	180	5700	1610.43	905.72

**Table 2:** Statistics of the parameters considered for colorectal cancer study group.

	N	Minimum	Maximum	Mean	Std. Deviation
Sex	49	1	2	1.55	0.50
Age (years old)	49	29	91	69.94	12.84
Hb (g/DL)	49	4.7	16	10.58	2.59
Ht (%)	49	17.1	46.9	33.11	6.64
PLT(/mm^3^)	49	139,000	792,000	340,551.00	129,012.30
WBC(/mm^3^)	49	3820	19,170	9127.14	3523.92
Neutr(/mm^3^)	49	2200	16,540	6627.14	3280.35
Lymph(/mm^3^)	49	177	3140	1530.35	607.70

**Table 3:** Confusion matrix for the ANN.

		PC (Predicted)	Non-PC (Predicted)
Training	PC (actual)	27 (TP)	9 (FN)
	Non-PC (actual)	3 (FP)	27 (TN)
Testing	PC (actual)	8 (TP)	5 (FN)
	Non-PC (actual)	10 (FP)	6 (TN)

**Table 4:** Confusion matrix for the SVM.

		PC (Predicted)	Non-PC (Predicted)
Training	PC (actual)	28 (TP)	8 (FN)
	Non-PC (actual)	9 (FP)	21 (TN)
Testing	PC (actual)	7 (TP)	6 (FN)
	Non-PC (actual)	2 (FP)	14 (TN)

**Table 5:** Confusion matrix for the RF.

		PC (Predicted)	Non-PC (Predicted)
Training	PC (actual)	36 (TP)	0 (FN)
	Non-PC (actual)	0 (FP)	30 (TN)
Testing	PC (actual)	10 (TP)	3 (FN)
	Non-PC (actual)	4 (FP)	12 (TN)

**Table 6:** Summary statistics for RF.

	Precision	Recall (or Sensitivity)	F1-Score	Accuracy	Specificity
ANN	0.44	0.61	0.651	0.48	0.38
SVM	0.77	0.53	0.63	0.72	0.88
RF	0.71	0.77	0.74	0.76	0.75

**Table 7:** Sensitivity values.

Input	Sensitivity Coefficient
Age	0.217220
Ht	0.153470
Hb	0.144125
Lymph	0.133404
Neutr	0.117887
WBC	0.112012
PLT	0.102972
Sex	0.018910

## Data Availability

The simulation files and data used to support the findings of this study are available from the corresponding author upon request.
